# Spatial Distributions, Characteristics, and Applications of Craniofacial Stem Cells

**DOI:** 10.1155/2020/8868593

**Published:** 2020-08-29

**Authors:** Geru Zhang, Qiwen Li, Quan Yuan, Shiwen Zhang

**Affiliations:** ^1^State Key Laboratory of Oral Diseases and National Clinical Research Center for Oral Diseases, West China Hospital of Stomatology, Sichuan University, Chengdu, China; ^2^Department of Oral Implantology, West China Hospital of Stomatology, Sichuan University, Chengdu, China

## Abstract

Stem cells play an irreplaceable role in the development, homeostasis, and regeneration of the craniofacial bone. Multiple populations of tissue-resident craniofacial skeletal stem cells have been identified in different stem cell niches, including the cranial periosteum, jawbone marrow, temporomandibular joint, cranial sutures, and periodontium. These cells exhibit self-renewal and multidirectional differentiation abilities. Here, we summarized the properties of craniofacial skeletal stem cells, based on their spatial distribution. Specifically, we focused on the *in vivo* genetic fate mapping of stem cells, by exploring specific stem cell markers and observing their lineage commitment in both the homeostatic and regenerative states. Finally, we discussed their application in regenerative medicine.

## 1. Introduction

The reconstruction of craniofacial bone defects is more challenging than that of the limb bone, as it requires both functional and esthetic recovery. Traditional therapies to regenerate craniofacial bone, including autologous bone grafts, allografts, and xenografts [1–3], exhibit different limitations and often fail to meet the demands of recovery [4–6]. Stem cell-guided regenerative medicine is an alternative that is currently the most promising approach to solve this problem.

Mesenchymal stem cells (MSCs) are groups of cells residing in different tissues and niches, such as the bone marrow, adipose tissue, teeth, and umbilical cord tissue. MSCs have been extensively used in tissue repair, organ reconstruction, immunomodulation, and even in the treatment of disease [7–11]. In addition, self-cell-constituted implantation results in reduced immunogenicity, and the molecules excreted from MSCs are beneficial for tissue recovery [12, 13]. The combination of MSCs with bioscaffolds further promoted MSC-based therapy by guiding MSC proliferation and migration [14].

To identify and isolate MSCs easily *in vitro*, the International Society for Cellular Therapy has proposed three criteria to define MSCs [15]. First, the isolated cells can adhere to plastic plates when cultured *in vitro*. Second, the cells express the CD73, CD90, and CD105 surface markers but not CD34, CD45, CD14 or CD11b, CD79a or CD19, and HLA-DR. Third, the cells can differentiate into osteocytes, chondrocytes, and adipocytes. In addition to *in vitro* characterization, the recent application and improvement of the fluorescent reporter mouse system and lineage tracing technique make the *in vivo* study of stem cells feasible [16]. Importantly, the *in vivo* study of stem cells can aid in accurately recapitulating the niche-dependent functions and interactions of stem cells.

MSCs from bones, including the bone marrow, periosteum, growth plate, and calvarium, have been the most thoroughly studied. It is now recognized that bone MSCs are highly heterogeneous populations that display variable self-renewal and differentiation potential. MSCs that commit to skeletal lineages and express selective surface markers (e.g., leptin receptor, PDGFR*α*, nestin, Cxcl12, Hox11, PTHrP, Sca1, Ctsk, Axin2, and Gli1) are now defined as skeletal stem cells (SSCs). Craniofacial SSCs are subgroups of cells residing in the calvarium, maxillary and mandibular bones, and tooth-supporting tissue. These cells display the basic characteristics of SSCs and are capable of self-renewal and multilineage differentiation. They can regenerate oral tissues and repair critical defects of craniofacial bones [17–20]. However, craniofacial SSCs are distinct from long bone SSCs, which might result from the different developmental origins and stem cell microenvironments/niches. The craniofacial bone originates from the mesoderm and neural crest, and the bony structure is formed by intramembranous ossification. Long bones, on the other hand, mainly originate from the mesoderm and are formed by endochondral ossification [20, 21]. In addition, the craniofacial bone is a flat bone with limited bone marrow, but the long bone is enriched with the bone marrow. Hematopoiesis-depleted or hematopoiesis-enriched-enriched environments result in totally different stem cell niches. Therefore, craniofacial SSCs are different from SSCs in long bones or other tissues. Interestingly, studies have shown that SSCs/MSCs from craniofacial bones exhibit superior osteogenic properties compared with long bone SSCs/MSCs in craniofacial tissue reconstruction [22–24]. A pioneering study also found that postnatal lineage-restricted craniofacial SSCs reverted to their embryonic plastic state and regained a neural crest cell phenotype in response to mandibular distraction for jaw regeneration [25]. Identifying subpopulations and illustrating the properties of craniofacial SSCs are thus crucial to stem cell-guided regenerative medicine.

In this review, we summarize the cranial and maxillofacial tissues in which stem cells reside as well as the characteristics of these stem cells and advancements in their applications.

## 2. Periosteum

**Figure 1:**
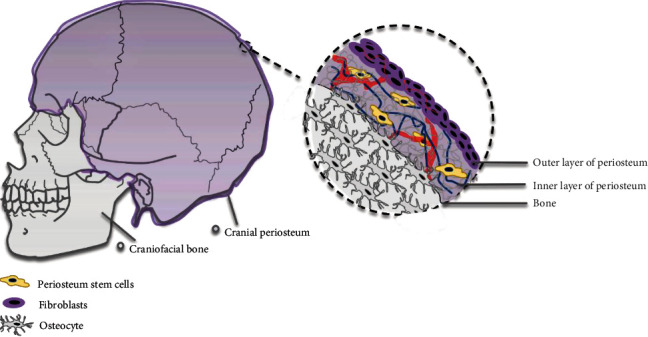
Stem cell distribution in the cranial bone. The stem cells are located in the inner layer of calvarium periosteum.

The surface of the bone is covered by the periosteum, which is a 50-150 *μ*m two-layer membrane with an abundance of nerves and blood vessels (Figure 1). The outer fibrous layer is adjacent to the surrounding soft fibrous and muscular tissue, while the inner layer is highly vascularized and provides a niche for progenitor cells [26, 27]. A large number of studies have already shown that precursor cells residing in the craniofacial bone periosteum play an important role in bone regeneration [28]. For a long time, no specific cell marker was available to identify and isolate craniofacial periosteum-derived stem cells (PSCs) [28, 29]. Recently, two specific surface makers (Ctsk^+^ and Mx1^+^*α*SMA^+^) have been identified.

Cathepsin K (CTSK) has long been regarded as a specific marker for osteoclasts. Using *Ctsk-mGFP* transgenic mice to trace cell lineages combined with single-cell RNA sequencing, Debnath et al. identified Ctsk^+^ periosteum stem cells as both long bone and calvarial periosteal skeletal stem cells (PSCs). Ctsk^+^ PSCs are capable of self-renewal, colony formation, and multilineage differentiation. Interestingly, Ctsk^+^ PSCs are highly plastic, as they can mediate not only intramembranous ossification but also endochondral ossification in response to bone injury [30]. In 2019, Park et al. observed that a group of postnatal long-term Mx1^+^*α*SMA^+^ periosteal stem cells contributed significantly to the injury repair of bone defects. In addition to being capable of self-renewal and clonal multipotency, Mx1^+^*α*SMA^+^ PSCs can migrate toward the injury site in response to a CCR5 ligand- (CCL5-) dependent mechanism, as visualized by *in vivo* real-time imaging of the calvarium [31].

## 3. Craniofacial Bone Marrow

Given that jawbones and teeth in the craniofacial system originate from the cranial neural crest, marrow stem cells in jawbones are considered to have characteristics different from those of long bone MSCs. Studies have been performed to compare the similarities and differences between stem cells in the craniofacial, axial, and appendicular regions. Human MSCs in the jawbone and iliac crest have been the most commonly studied, as these sites are ideal for marrow aspiration. Akintoye et al. cultured jawbone MSCs and iliac crest MSCs from the same individual and found that jawbone MSCs displayed a higher proliferation rate, delayed senescence, and greater differentiation potential. *In vivo* transplantation results showed that jawbone MSCs formed more bone, whereas iliac crest MSCs formed more compacted bone along with hematopoietic tissue [32]. Using tube formation assays and 3D fibrin vasculogenic tests, Du et al. found that jawbone MSCs showed stronger angiogenic propensities than iliac crest MSCs when they were cocultured with human umbilical vein endothelial cells (HUVECs). Coculture with jawbone MSCs allowed HUVECs to form more tube-like structures *in vitro* and larger vessels *in vivo* [33]. The increase in the expression of the basic fibroblast growth factor (bFGF) by jawbone MSCs is the key factor contributing to angiogenesis. However, the chondrogenic and adipogenic potential of jawbone MSCs is weaker than that of iliac crest MSCs [34, 35].

Several populations of SSCs in the long bone marrow were identified, including leptin-receptor-expressing (LepR^+^) SSCs, nestin-expressing (Nestin^+^) SSCs, Gremlin 1-expressing (Grem1^+^) SSCs, glioma-associated oncogene 1-expressing (Gli1^+^) SSCs, and CD45^−^Ter^−^119^−^Tie2^−^AlphaV^+^Thy^−^6C3^−^CD105^−^CD200^+^ SSCs [36–39]. However, their identity and function in the craniofacial bone remain unclear. We recently identified a quiescent population of tissue-resident LepR^+^ SSCs in jawbone marrow that became activated in response to tooth extraction and contributed to intramembranous bone formation [40]. Using *LepR-Cre*; *tdTomato*; *Col2.3-GFP* reporter mice, we found that these LepR^+^ cells remained quiescent in the physiological state and gradually increased in activity with age. External stimuli such as tooth extraction activated LepR^+^ SSCs, which rapidly proliferated and differentiated into Col2.3-expressing osteoblasts, contributing significantly to extraction socket repair. Ablation of LepR^+^ SSCs with diphtheria toxin dramatically impaired the bone healing process. A mechanistic study showed that alveolar LepR^+^ SSCs are responsive to parathyroid hormone/parathyroid hormone I receptor (PTH/PTH1R) signaling. Knockout of *Pth1r* in the LepR^+^ cell lineage disrupted the bone formation process.

## 4. Temporomandibular Joint

The temporomandibular joint (TMJ) is located between the temporal bone and the mandible. It is one of the most frequently used joints in humans and is the only diarthrosis in the stomatognathic system. The occurrence of TMJ osteoarthritis is highly prevalent in humans, yet the regenerative capacity of condylar cartilage is limited. Therefore, identifying and isolating stem cells in the TMJ is crucial for osteoarthritis amelioration and regeneration.

**Figure 2:**
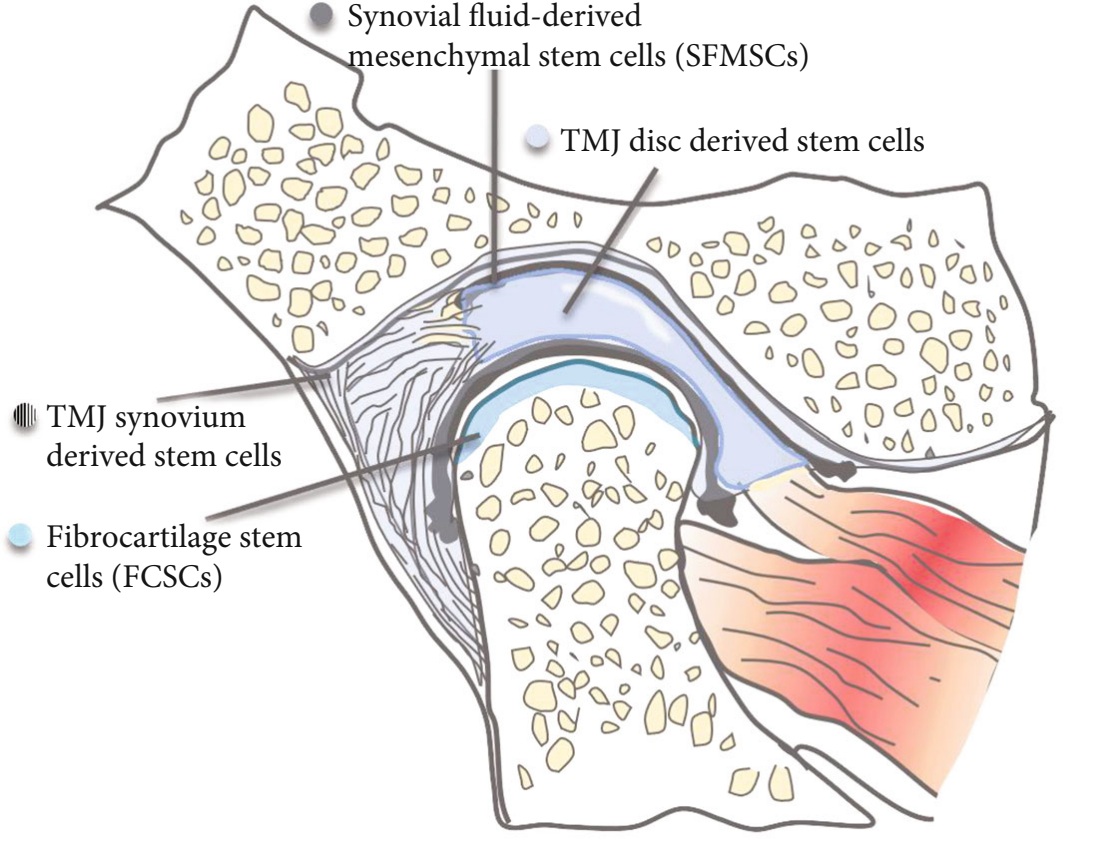
Stem cell distribution in TMJ. Stem cells are located in the synovial fluid, synovium, disc of TMJ, and surface zone of condylar cartilage.

Two types of stem cells reside in the TMJ (Figure 2). One type is TMJ synovium-derived stem cells, and the other type is fibrocartilage stem cells (FCSCs). In 2011, Liu et al. isolated and cultured stem cells from human TMJ synovial fluid and found that these cells exhibit fibroblastic and spindle shapes. Flow cytometry analysis showed that these cells express MSC markers and could be induced to differentiate toward osteogenic, chondrogenic, adipogenic, and neurogenic lineages. Thus, these synovium-derived cells are stem cells [41]. Koyama et al. found STRO-1- and CD146-expressing stem cells in the TMJ synovial fluid of patients with temporomandibular joint disorder. These cells showed great potential to differentiate into chondrocytes, osteoblasts, adipocytes, and neurons [42]. Stem cells were also isolated from the radiolucent zone of TMJ ankylosis patients, but they had a slower proliferation rate and lower osteogenic differentiation capacity than BMSCs [43]. In 2014, Sun et al. isolated synovial fragment cells from the synovial fluid of temporomandibular disease patients and revealed the multilineage differentiation capacity of this group of cells [44]. Fibrocartilage stem cells (FCSCs) reside in the superficial zone of condylar cartilage. A single FCSC could generate a cartilage anlage, which then undergoes autogenous bone formation and supports a hematopoietic microenvironment. Wnt signaling impairs the FCSC niche and results in cartilage degeneration. Intra-articular injection of the Wnt inhibitor sclerostin reconstructed the stem cell niche and repaired TMJ injury, indicating a potential therapeutic strategy for patients with fibrocartilage defects and disease [45].

## 5. Sutures

**Figure 3:**
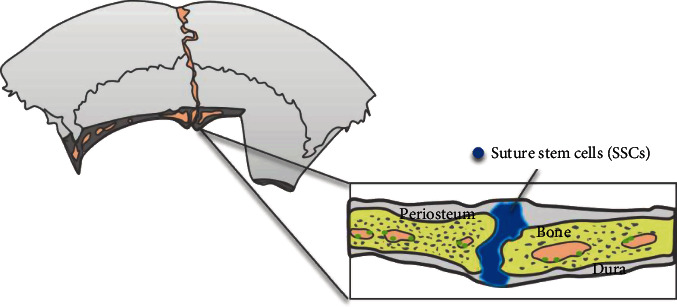
Stem cell distribution in sutures.

In summary, the flexible connection between paired calvarial bones permits the deformation of the skull during birth, directs the growth of the skull, and acts as a shock absorber that can cushion the load of mastication (Figure 3). Humans and mice both have four sutures. Metopic sutures (called interfrontal sutures in mice) and sagittal sutures are vertically distributed, and the osteogenic fronts about each other. Coronal sutures and lambdoid sutures are horizontally distributed, and their osteogenic fronts overlap with each other. Unossified sutures are recognized as the bone growth center of the postnatal skull vault [46], where the new bone precipitates at the edges of the bone front. Premature closure of the suture could lead to craniosynostosis. Studies have demonstrated a unique stem cell niche in cranial sutures, where multiple populations ofSeveral subpopulations of suture mesenchymal stem cells (SuSCs) were identified, including Gli1-positive (Gli1^+^) cells, Axin2-expressing (Axin2^+^) cells, and postnatal Prx1-expressing (Prx1^+^) cells [46–49]. All these cells possess the ability for self-renewal and continually produce skeletal cell descendants. Clonal expansion analysis demonstrated that SuSCs were capable of forming bones during calvarial development. SuSCs expand dramatically in the damaged site and contribute directly to skeletal repair; the nearer the cells are to the sutures, the better the recovery is [46, 50]. However, the spatiotemporal properties of SuSCs differ. During the early stage of postnatal development, Gli1^+^ cells were distributed throughout the periosteum, dura, and sutures, whereas Axin2^+^ cells and Prx1^+^ cells appeared only in the sutures. At 4 weeks of age, all of the SuSCs were restricted to the sutures. Gli1^+^ SuSCs were capable of trilineage differentiation into chondrocytes, osteoblasts, and adipocytes; Axin2^+^ SuSCs mainly gave rise to the chondro- and osteolineages, whereas Prx1^+^ SuSCs could only differentiate into osteoblasts. Depletion of Gli1^+^ or Axin2^+^ cells but not Prx1^+^ SuSCs caused craniosynostosis. Additional comparisons of the three types of cells are listed in Table 1.

## 6. Periodontium

**Figure 4:**
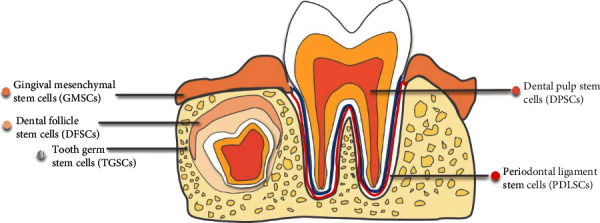
Stem cell distribution in dental tissues. Stem cells are located in gingiva, dental follicle, tooth germ, dental pulp, and periodontal ligament.

The tooth is a hard tissue containing a vascularized and nerve-rich pulp chamber and is surrounded by the tooth-supporting periodontium, including the periodontal ligament, cementum, and alveolar bone. Multiple stem cell niches exist in dental tissues, as teeth are uniquely shaped and are subject to complicated microenvironments with occlusal force and microorganisms [53]. To date, more than seven kinds of dental stem cells have been found, including dental pulp stem cells (DPSCs) [54], human exfoliated deciduous teeth (SHED) [55], periodontal ligament stem cells (PDLSCs) [56], dental follicle progenitor cells (DFPCs) [57], stem cells from dental apical papilla (SCAP) [58], tooth germ stem cells (TGSCs) [59], gingival mesenchymal stem cells (GMSCs) [60], and human natal dental pulp stem cells (NDP-SCs) (Figure 4) [61]. All of them are capable of self-renewal, proliferation, and multidirectional differentiation [62]. Among them, PDLSCs are the only kind of stem cells that can differentiate into osteoblasts *in vivo* and contribute to the construction of alveolar bone and tooth extraction sockets.

As reported in 2004, Seo et al. isolated and identified PDLSCs from human impacted wisdom teeth for the first time [56]. In addition to wisdom teeth, PDLSCs can be extracted from permanent tooth root surfaces [63], deciduous tooth [64–66], or even inflammatory periodontal tissues [67]. However, PDLSCs derived from different environments display different properties related to proliferation and osteogenic potential. For example, studies have found that PDLSCs from deciduous teeth promote osteoclastogenesis and lead to root absorption [68]. PDLSCs from inflammatory tissue are predisposed to a pathological local microenvironment [69]. The regulatory mechanism of PDLSC biological behavior remains to be revealed.

To exploit the osteogenic potential of PDLSCs, the osteogenic mechanism of PDLSCs needs to be clarified. It has been reported that antidifferentiation noncoding RNA (ANCR) [70], long noncoding RNAs (lncRNAs) [71], and microRNA-182 and microRNA-214 [72, 73] regulate the proliferation and osteogenic differentiation of PDLSCs. He et al. [74] and Yan et al. [75] found that hypoxia and cannabinoid receptor I (CB1) could alter the activity of PDLSCs through the p38/MAPK pathway. Meanwhile, the PI3K-AKT-mTOR pathway [76] and NF-*κ*B axis [77] are also involved in the modulation of PDLSCs. In clinical practice, additional topics, such as how metformin contributes to the osteogenic potential of PDLSCs [78] and how nicotine [79, 80] and *Porphyromonas gingivalis* [81] weaken the osteogenic potential of PDLSCs, remain to be investigated.

PDLSCs exhibit high potential for tissue regeneration and are capable of giving rise to osteoblast-/cementoblast-like cells, adipocytes, chondrogenic cells, neurogenic lineage cells, endothelial cells, cardiac myocytes, and Schwann cells *in vitro* [62]. For stem cell identity and fate commitment, Roguljic and colleagues identified *α*SMA as a marker of PDLSCs *in vivo* [82]. *α*SMA^+^ PDLSCs expanded over time and mainly gave rise to cells in the apical region. Following periodontal ligament injury, PDLSCs proliferated and generated mature cementoblasts, osteoblasts, and fibroblasts within the periodontium. However, *α*SMA^+^ PDLSCs only made minor contributions to periodontium homeostasis and repair. Yuan and colleagues used Axin2 to track progenitor cells in the periodontal ligament and reported that PDLSCs are responsive to Wnt signaling [83]. Axin2^+^ PDLSCs remain quiescent under physiological conditions and differentiate into osteoblastic cells for alveolar bone repair when tooth extraction injury occurs. Most recently, using genetic fate mapping, Men et al. identified Gli1^+^ PDLSCs in adult mouse molars that gave rise to periodontal ligament, alveolar bone, and cementum in both the homeostatic state and during injury repair [84]. Gli1+ PDLSCs are enriched in the apical tooth region and surround the neurovascular bundle, and they are activated by canonical Wnt signaling. Sclerostin secreted by alveolar bone osteocytes inhibits Wnt signaling. Occlusal force can inhibit sclerostin secretion. Therefore, a feedback loop that regulates stem cell activities is present in the stem cell niche, where occlusal force-mediated inhibition of sclerostin secretion by alveolar bone osteocytes promotes Gli1^+^ cell maintenance and activation. The authors also compared other stem cell markers with Gli1. They concluded that labeling of Gli1 more efficiently identified PDLSCs compared to labeling of *α*SMA, LepR, NG2, and Pdgfr*α*. Using an inducible Cre system and immunostaining, they also reported that LepR^+^, NG2^+^, and Pdgfr*α*^+^ cells are descendants of Gli1+ cells in the periodontal ligament.

## 7. Application of Craniofacial Stem Cells

Stem cells for tissue regeneration have been widely exploited and are mainly applied in three ways: direct transplantation of a specific type of stem cells, combined application of different types of stem cells, and the use of stem cells in combination with biological scaffolds. However, the regenerative techniques used in the long bone cannot be easily extended to craniofacial applications because the microenvironment of craniofacial tissue is quite different from that of the long bone. Oral pathological factors (e.g., microorganisms, nicotine, and bisphosphonate) greatly affect the biological behavior of craniofacial stem cells. For instance, Kim et al. found that excessive nicotine intake will induce the vacuolation of jawbone MSCs and impair their proliferation and differentiation capacity [85]. Akintoye et al. found that jawbone MSC self-renewal and proliferation ability were impaired when the MSCs were treated with bisphosphonate, which might be associated with the pathogenesis of bisphosphonate-associated osteonecrosis. Therefore, to minimize potential damage, the use of appropriate ways to amplify and induce stem cells to differentiate toward the osteoblast lineage is particularly important. Wang et al. reported that vitamin C and vitamin D were ideal stimulants of craniofacial PDLSCs for osteoblast differentiation *in vitro* [26]. Naung et al. proposed a protocol to cultivate palate periosteum-derived MSCs in serum-free and xeno-free medium, which could become a useful source of MSCs for clinical applications [86]. Moreover, a recent study found that induced pluripotent stem cells (iPSCs) generated from human jaw periosteum cells expressed MSC markers and possessed strong mineralization ability [87].

The application of MSCs for regenerative medicine should be performed with caution because the stem cells obtained and expanded with plastic culture are highly heterogeneous and might not be intrinsically multipotent [88]. It should be noted that the minimal criteria, including adherence to the plastic plate, induction of multidirectional differentiation, and detection of appropriate surface marker profiles, are not sufficient to identify *bona fide* stem cells [89]. Some quiescent stem cells might not readily adhere to the plastic, and the heterogeneous cell mixture contains lineage-committed stem cells that give rise to cells of native tissue origin. For instance, bone marrow stem cells show an intrinsic propensity for differentiation toward osteolineages [90]. The surface markers of bone marrow stem cells are specific for fibroblast-like cells rather than stem cells [88]. Therefore, *in vivo* clonal assays and fate mapping are essential to identify *bona fide* stem cells [38]. Future studies are warranted to identify the stem cell properties of craniofacial stem cells, which will benefit clinical applications.

## 8. Conclusions

This review describes the stem cells found in the craniofacial periosteum, craniofacial bone marrow, TMJ, cranial suture, and periodontal ligament. Craniofacial stem cells express specific cell surface determinants, possess a low self-renewal rate, and show multidifferentiation ability. They rapidly proliferated and differentiated into osteoblasts in response to injury. In addition, craniofacial tissues are easily obtained from human jawbones with minimal invasiveness when clinicians perform implant surgeries, tooth extractions, and periodontal surgeries. All these results indicated that craniofacial stem cells are an ideal resource for tissue engineering.

However, the current research on craniofacial stem cells is still inadequate and lacks depth. Specific cell markers for the isolation of stem cells are still lacking. *In vivo* clonal assays and lineage fate mapping are warranted in future studies, which could facilitate the therapeutic application of craniofacial stem cells *in vivo* and in the clinic.

## Figures and Tables

**Table 1:** The subpopulations of suture mesenchymal stem cells and their characteristics.

Cell types		Gli1^+^ cells [50, 51]	Axin2^+^ cells [46]	Postnatal Prx1^+^ cells [52]
Distribution	Early stage	All over the periosteum, dura, and the craniofacial sutures	In the calvarial sutures	In the calvarial sutures
	One month after birth	Self-renewal	Only in the sutures	/
Stemness	Self-renewal		Slow-cycling cells	
	Contribution to other tissues	Suture mesenchyme, periosteum, dura mater, and parts of the calvarial bones	Suture mesenchyme and bone matrix near the osteogenic fronts	All calvarial tissues, except bone marrow osteoblasts
	Ability to repair the defect	Unequivocal and potentially exclusive contribution of the sutural mesenchyme to calvarial injury repair		
Ablation		Craniosynostosis	Craniosynostosis	Did not result in craniosynostosis or any other major craniofacial phenotype
MSC markers		CD90, CD73, CD44, Sca1, and CD146	LepR	Pdgfr*α* and Mcam/CD146 (upregulation), Ccne2, Mcm4, and Pcna (downregulation), Itga2, Itga3, and Itga6
Differentiation	Osteoblasts	+	+ (upon external stimulation)	+ (stimulated with recombinant WNT3A)
	Chondrocytes	+	+ (upon external stimulation)	/
	Adipocytes	+	/	/
Foundation of each study		Gli1 is the master transcriptional factor of hedgehog signaling and is indispensable for bone development and homeostasis. Gli1+ stem cells have been identified in canine and long bones.	Axin2 plays an irreplaceable role in the Wnt, BMP, and FGF signaling pathways; Axin2 knockout mice showed craniosynostosis.	Prx1 was previously shown to be highly expressed during limb bud formation and craniofacial development.
